# Language equivalence of the modified falls efficacy scale (MFES) among English- and Spanish-speaking older adults: Rasch analysis

**DOI:** 10.1186/s12877-020-01627-3

**Published:** 2020-08-12

**Authors:** Robert J. Lucero, Sergio Romero, Robert Fieo, Yamnia Cortes, Jeannie P. Cimiotti, Lusine Poghosyan

**Affiliations:** 1 Department of Family, Community, and Health System Science, Center for Latin American Studies, College of Nursing, University of Florida, 1225 Center Drive, Gainesville, Florida 32610 USA; 2 North Florida/South Georgia Veterans Health System, Center of Innovation on Disability and Rehabilitation Research, 300 E. University Avenue, Gainesville, FL 32601 USA; 3 Department of Epidemiology, College of Public Health and Health Professions, University of Florida, 1225 Center Drive, Gainesville, Florida, NY 32610 USA; 4 The University of North Carolina at Chapel Hill, School of Nursing, S. Columbia Street, Chapel Hill, NC 27599 USA; 5 Department of Family, Community, and Health Systems Science, Nell Hodgson Woodruff School of Nursing, Emory University, 1520 Clifton Road, NE, Atlanta, GA 30322 USA; 6 Columbia University, School of Nursing, Center for Health Policy, 560 W. 168th Street, New York, NY 10032 USA

**Keywords:** Fear of falling, Rash analysis, Language equivalence, Psychometric measurement, Modified falls efficacy scale

## Abstract

**Background:**

To investigate item-level measurement properties of the Modified Falls Efficacy (MFES) Scale among English- and Spanish-speaking urban-dwelling older adults as a means to evaluate language equivalence of the tool.

**Methods:**

Secondary analysis of survey data from 170 English (*n* = 83) and Spanish (*n* = 87) speaking older adults who reported to the emergency department of a quaternary medical center in New York City between February 2010 and August 2011. The Rasch rating scale model was used to investigate item statistics and ordering of items, item and person reliability, and model performance of the Modified Falls Efficacy Scale.

**Results:**

The Modified Falls Efficacy Scale, for English- and Spanish-speakers, demonstrated acceptable fit to the Rasch model of a unidimensional measure. While the range of the construct is more limited for the Spanish group, the interval between tasks are much closer, reflecting little to no construct under-representation.

**Conclusion:**

There is rationale for continued testing of a unidemsional English- and Spanish-MFES among urban community-dwelling older adults. Large-scale international studies linking the unidemsional MFES to patient outcomes will support the validity of this tool for research and practice.

Twenty-eight to 35 % of adults worldwide who are 65 years of age and older fall each year [1]. Falls are a leading cause of emergency department (ED) visits and long-term functional impairments. Hospitalization due to falls varies however the average length of stay is longer for falls than other injuries, and ranges from 4 to 15 days in Switzerland, Sweden, United States (US), Western Australia, Province of British Columbia and Quebec in Canada [1]. In addition to the physical consequences associated with falling, psychological distress characterized as loss of confidence, low perceived self-efficacy, and fear has been identified in both persons with and without a history of falling [2–5].

## Background

Fear of falling is a common concern among community-dwelling older adults [6]. First introduced in the literature in the early 1980s, fear of falling was defined as the intense fear of standing or walking after an accidental fall [7]. It was later defined as ongoing concern about falling and avoidance of activities that an individual is capable of performing [8]. Fear of falling is associated with a reduction in activities of daily living and quality of life [9–11]. More than 30% of community-dwelling older adults fear falling, and nearly half restrict the amount or type of activities they perform because of fear [12–16].

Measures have been developed to assess the psychological aspects of falling such as fear, self-efficacy, loss of confidence, concern, and activity avoidance [17–20]. The Falls Efficacy Scale (FES) can be used to assess an individual’s confidence in performing common activities of daily living without falling. The focus of the FES is on activities that could be difficult for individuals who fear falling due to functional impairment [21]. The FES has limited usefulness among higher functioning community-dwelling older adults who may have greater confidence in performing the activities. Others have attempted to improve upon the FES by including difficult and social activities in a measure known as the FES – International [22]. While the results yielded what appears to be a reliable modification of the FES, “the FES – International actually assesses ‘concern’ about falling” and not fear of falling [22] (p.617). The distinction between these two latent constructs (i.e., concern about falling and fear of falling) has implications for an accurate representation of fear of falling among older adults [3].

To preserve the intended use of the FES, Hill and colleagues developed the 14-item Modified Falls Efficacy Scale (MFES) which includes “tasks commonly reporter by fallers as inducing greater fear of falling” [23] (p. 1026). In addition to activities in the FES, an individual’s confidence in using public transportation, hanging out the wash/gardening, crossing roads, and using steps at home are included in the MFES. The MFES is associated with activities of daily living, social participation, gait speed, and stride length [24, 25]. The MFES has an acceptable internal consistency reliability (Cronbach’s alpha = 0.95) and test-retest reliability, 0.95 [23]. Discriminant validity was established comparing healthy older adults to individuals from a Falls Balance Clinic; significantly lower falls-efficacy scores were observed in the clinic-based group [26]. The MFES appears to be a valid and reliable measure of fear of falling and demonstrated utility in falls risk assessment.

Because the MFES has only been validated with Australian, French, and Serbian populations, it is essential to determine the tool’s ability to characterize an individual’s self-perceived fear of falling among community-dwelling older adults in the US [23, 27, 28]. With a fast growing diverse older adult population in the US, there is urgency for a valid and reliable clinical tool to assess fear of falling when individuals perform common indoor and outdoor activities. With older Hispanic adults projected to grow from under 3 million to 17.5 million by 2050, many of whom only speak Spanish, there is a need to accurately characterize fear of falling among Spanish-speaking older adults living in the US [29].

## Methods

### Aim

We investigated item-level measurement properties of the MFES among English- and Spanish-speaking community-dwelling older adults in the US as a means to evaluate language equivalence of the tool.

### Study design

This was a secondary analysis of cross-sectional data collected between February 2010 and August 2011 for a study that explored the predictive validity of two falls screening methods: (1) the report of a fall that precipitated an ED visit and (2) ambulatory-care patient’s report of a fall in the past year. The current study included the entire sample of the parent study and analyzed the data with a focus on what measurement properties could be revealed about the MFES among English- and Spanish-speaking community-dwelling older adults in the US. Institutional Review Board of the Columbia University Medical Center approved the conduct of this study.

### Sample

In the parent study, a research nurse screened all potential community-dwelling older adult participants. Participants who fell self-reported to the ED after a fall at home or in the community. Participants who did not fall were in the ED for any other reason than a self-reported fall. Individuals were recruited in the parent study if they were at least 55 years of age, spoke English or Spanish, admitted to the ED, and discharged home with a non-serious injury. Participants whose fall resulted in abrasions and contusions were included if they were discharged home. Participants with a diagnosis of cognitive impairment were not included in the primary study. The research nurse reviewed the medical record to confirm that the participant did not have cognitive impairment.

### Data collection

After a potential participant was determined eligible in the parent study, the research nurse obtained informed consent and administered the MFES and a demographic questionnaire in the participant’s primary language. Data from 83 English-speaking and 87 Spanish-speaking participants from the parent study are included in the current study.

### The MFES

The MFES elicits responses to the question, “How confident/sure are you that you do each of the activities without falling?”, for 14 items (see Table 2). Using a visual analog scale items are scored from 0 to 10 (0 - “not confident/not sure at all”; 5 - “fairly confident/fairly sure”; 10 - “completely confident/completely sure”) with a total score of 0–140.

### Translation of the MFES

A bilingual investigator whose primary language is Spanish, was born in the Dominican Republic, and is a member of the populations included in this study, ensured the cultural relevance of the MFES items for the Dominican and Puerto Rican participants represented largely in the primary study location (i.e., New York City). A translator from the Spanish Translation Center of the sponsoring organization’s Clinical Trials Office whose primary language is Spanish translated the MFES for the primary study. The goals of the Spanish Translation Center are to: 1) ensure precise linguistic meaning of Spanish language materials for valid data collection and comparisons across languages, and 2) protect the rights and welfare of research participants by producing translations that reflect the accuracy of ideas expressed in English. The Spanish Translation Center forward-translation focused on conceptual rather than literal translations, and the use of natural and acceptable language among Dominican and Puerto Rican individuals [30]. Spanish items were back-translated into English by a bilingual investigator whose primary language is English. As with the forward-translation, the back-translation focused on conceptual and cultural and not linguistic similarity. A copy of the translated version of the MFES used in this study is available upon request from the corresponding author.

### Statistical analysis

The factor structures of the English (*n* = 83) and Spanish (*n* = 87) MFES were investigated separately using exploratory factor analysis (EFA). A sample size of at least 50 and not more than 100 respondents is adequate to evaluate the psychometric properties of social constructs such a fear of falling [31]. Weighted least squares mean and variance (WLSMV) estimation was conducted on the English and Spanish samples. Item responses were treated as categorical variables with values of 0, 5, and 10. The number of factors were determined based on a visual inspection of the scree plot, eigenvalues > 1.0, and a ratio of the first to second eigenvalue greater than four [32–34]. The chi square (χ^2^) and goodness-of-fit indices (i.e., comparative fit index [CFI], Tucker–Lewis Index [TLI], root mean square error of approximation [RMSEA], and standardized root mean square residual [SRMR]) were calculated for each factor solution [35, 36]. Oblique rotations using the GEOMIN method were generated to assist in factor interpretation. The final factor solution and interpretation was based on model fit using the CFI, TLI, RMSEA, and SRMR.

The Rasch rating scale model was used to investigate item statistics and ordering of items, item and person reliability, and model performance. Rasch analysis is a modern test theory measurement-model in contrast to classical test theory. Modern test theory can provide information beyond classical test theory metrics (e.g., factor analysis, alpha reliability). For example, Cronbach’s reliability coefficient (i.e., classical test theory), provides an average estimate for the entire sample. Modern test theory provides an information function in which reliability can be estimated separately along different levels of the trait under investigation.

Rasch analysis can convert ordinal level raw questionnaire scores into more precise interval level measurement [37]. Rasch can also be used to confirm or establish a formal hierarchy beyond averages of item/task difficulty. The invariant item ordering property goes beyond merely ordering subjects by a mean score. Rasch achieves these gains over classical test theory approaches by estimating both person ability and item difficulty. An individual’s response to a particular item is based on individual parameters (e.g., ability or any latent traits) and characteristics of item parameters (e.g., test difficulty).

Item level psychometric properties of the English- and Spanish-MFES were evaluated separately using a Rasch rating scale model. Tests of fit were used to examine the difference between observed response and that expected by the model. Appropriate fit means that items are internally consistent and the interval-level locations or scores can be used for statistical analyses including comparing mean locations of groups. In WINSTEPS, these scores are called INFIT and OUTFIT statistics. INFIT represents the difference between observed and expected responses for items that have a difficulty level near the person’s ability level. OUTFIT includes the differences for all items, irrespective of how far away the item difficulty is from the person’s ability [38]. The extent to which the 14 items of the MFES fit the fear of falling was assessed by goodness-of-fit statistics, where a good INFIT and OUTFIT mean-square (MNSQ) can range from 0.5 to 2.0 [39].

Item and person reliability and variance explained by the Rasch measurement model were evaluated using Fischer’s instrument quality criteria [39]. Participant’s ability versus item difficulty was assessed using a person-item map to identify floor-and-ceiling effects of the MFES. To determine equivalence of the English- and Spanish-MFES, we compared the hierarchical structure of the two versions to evaluate the range and precision of a 14-item unidimensional scale.

All analyses were computed using Statistical Package for the Social Sciences (SPSS) v.22, Winsteps, and Mplus 5.1.

## Results

English-speakers on average were slightly older (69.2 vs 66.4 years), and a larger percent were female (77.1% vs 59.8%) and reported falling (61.4% vs 55.2%) compared to Spanish-speakers (see Table 1). More than a majority of Spanish-speakers reported being Hispanic while 18.1% of the English-speakers reported being Hispanic. A larger percent of Spanish-speakers (42.5%), when compared to English-speakers (16.8%), self-identified belonging to a race other than White or Black/African American. A larger percent of Spanish-speakers had less than a high school education when compared to English-speakers (67.8% vs 26.4%). Slightly more than half of all participants reported beeeing windowed, divorced, or separated.

**Table 1:** Comparison of demographic characteristics of English- and Spanish-speaking participants (*N* = 170)

**Variable**	**English** ***n*** **= 83 (48.8%)**^**b**^	**Spanish** ***n*** **= 87 (51.2%)**^**b**^
Age (mean, sd)	69.2, 10.7	66.4, 8.5
Faller	51 (61.4%)	48 (55.2%)
Female	64 (77.1%)	52 (59.8%)
Hispanic	15 (18.1%)	86 (98.9%)
Race		
White	36 (43.4%)	11 (12.6%)
Black/African American	29 (34.9%)	4 (4.6%)
Other^a^	14 (16.8%)	37 (42.5%)
Marital Status		
Married	22 (26.5%)	31 (35.6%)
Widowed/Divorced/Separated	44 (53.0%)	46 (52.8%)
Never married	14 (16.9%)	8 (9.2%)
Education		
No Schooling	2 (2.4%)	6 (6.9%)
Grade School	20 (24.0%)	53 (60.9%)
High School	17 (20.5%)	12 (13.8%)
College	43 (51.7%)	13 (14.8%)

*sd* Standard deviation

^a^Other = American Indian/Alaska Native, Asian, and Other

^b^Not all categories add to 100% due to no response or refused to answer item

### Exploratory factor analysis

Based on the scree plot of the MFES-English, one factor explained most of the variability in the data. This factor yielded an eigenvalue of 12.76 with eigenvalues of the second and third factors below 1.0 (i.e., .392 and .298). The scree plot of the MFES-Spanish revealed that one factor explained most of the variability in the data with an eigenvalue of 11.43 and second and third factors of eigenvalues equal to .643 and .453. The goodness-of-fit statistics for the MFES-English were χ^2^ = 74.798 (df = 77, *P* > 0.54), CFI = 1.00, TLI = 1.00, RMSEA = .00, and SRMR = 0.036. The goodness-of-fit statistics for the MFES-Spanish were χ^2^ = 110.765 (df = 77, *P* < 0.008), CFI = .994, TLI = .993, RMSEA = .071, and SRMR = .050. English- and Spanish-MFES item communalities met a minimum threshold of 0.40, and are available upon request of the author [40].

### Rasch analysis

Table 2 represents item- and person-level psychometric properties of the English- and Spanish-MFES. For the English version, “good” INFIT and OUTFIT MNSQ values ranged from 0.51 to 1.79 with a “fair” OUTFIT MNSQ value of 0.35 for *Walk inside house* (columns 4 and 5). On the other hand, “good” INFIT and OUTFIT MNSQ values for the Spanish version ranged from 0.51 to 1.74 with a “poor” OUTFIT MNSQ value of 2.86 for *Answer door/telephone* (columns 9 and 10). All but one item in both versions of the MFES were within acceptable INFIT and OUTFIT MNSQ values. Both versions of the MFES obtained similar item and person reliability, model variance, and item and person separation indices (rows 16, 17 and 18). Both versions exhibited “fair” item reliability and “good” person reliability [39]. The closeness of the percentages for the variance explained by the Rasch measurement model indicates that the English- and Spanish-MFES appear to have “very good” quality of measurement properties [39].

**Table 2:** English- and Spanish-MFES Item Functioning

**English (*n*** **= 83)**					**Spanish (*n*** **= 87)**				
***Items***	**Measure**	SE*	**INFIT MNSQ**^**†**^	**OUTFIT MNSQ**	***Items***	**Measure**	**SE**	**INFIT MNSQ**	**OUTFIT MNSQ**
*Use steps at home*	2.43	0.33	1.18	1.18	*Use steps at home*	1.63	0.27	0.91	0.90
*Use public transportation*	1.59	0.34	1.79	1.59	*Reach in cabinets/closet*	1.26	0.27	1.07	1.02
*Cross roads*	1.20	0.33	0.97	0.96	*Gardening/hanging out wash*	1.23	0.28	0.55	0.51
*Gardening/hanging out wash*	1.16	0.36	1.03	0.84	*Cross roads*	0.82	0.27	1.30	1.14
*Simple shopping*	0.60	0.35	0.91	0.70	*Simple shopping*	0.51	0.28	0.96	0.90
*Reach in cabinets/closet*	0.07	0.36	1.37	1.24	*Use public transportation*	0.36	0.28	1.18	1.59
*Take a bath/shower*	−0.08	0.36	0.76	0.51	*Light housekeeping*	0.04	0.29	1.09	0.84
*Light housekeeping*	−0.18	0.37	0.86	0.75	*Get in/out of chair*	− 0.38	0.30	0.58	0.51
*Get in/out of chair*	−0.35	0.37	0.84	0.94	*Take a bath/shower*	−0.65	0.30	0.94	1.33
*Prepare a simple meal*	−0.80	0.40	0.81	0.69	*Prepare a simple meal*	−0.75	0.31	1.01	0.70
*Get in/out of bed*	−0.96	0.40	0.75	0.71	*Get in/out of bed*	−0.84	0.31	0.76	0.65
*Walk inside house*	−1.12	0.41	0.53	0.35	*Get dressed/undressed*	−0.94	0.31	0.85	0.95
*Get dressed/undressed*	− 1.30	0.42	1.20	1.25	*Answer door/telephone*	−1.04	0.32	1.74	2.86
*Answer door/telephone*	−2.25	0.51	0.83	1.37	*Walk inside house*	−1.25	0.32	1.33	1.08
**Item/Person Reliability**	0.89 / 0.77				**Item/Person Reliability**	0.89 / 0.76			
**Model Variance** **(obs/exp)**^**ǂ**^	76.3% / 75.7%				**Model Variance (obs/exp)**	75.1% / 71.2%			

*SE Standard error, ^**†**^*MNSQ* Mean-square, ^**ǂ**^*obs/exp*. Observed/expected

**Fig. 1:**
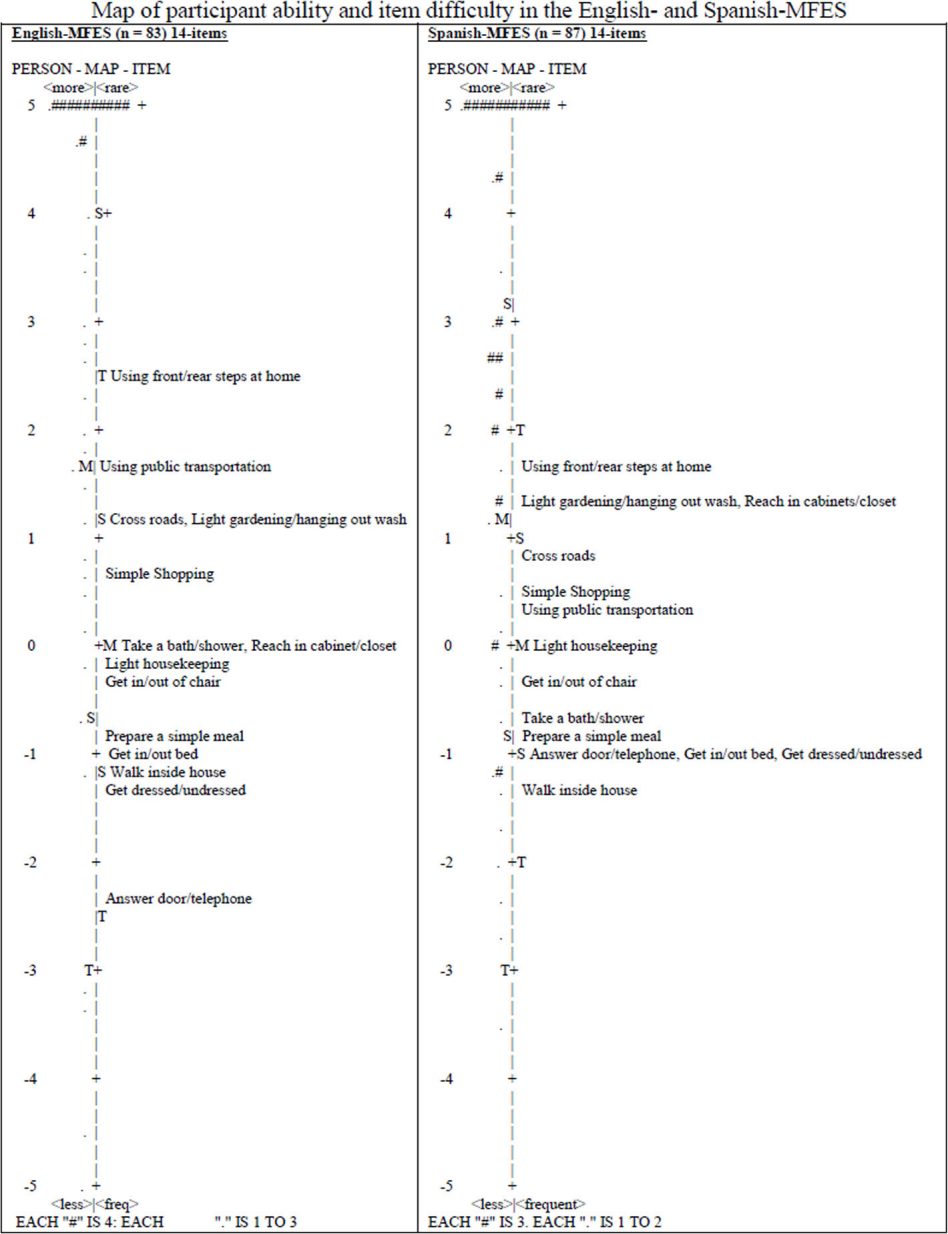
Map of participant ability and item difficulty in the English- and Spanish-MFES

Figure 1 illustrates the English- and Spanish-MFES participant ability versus item difficulties map. The English-MFES items are more dispersed around the middle space (i.e., 0 logits) than the Spanish-MFES items. While the *Light housekeeping* item occupies the middle space in both versions of the MFES, the middle space of the English-MFES is also occupied by *Take a bath/shower, Reach in cabinet/closet, and Get in/out of chair*. The hierarchical order of the items differed for both versions of the scale but all disordered items are within 1 or 2 standard errors of their counterpart and cannot be considered statistically different (see Table 2). The participant distributions, shown using the pound and period symbols, are clustered above the scale distribution of both versions. This clustering represents a ceiling effect for the English- and Spanish-speaking participants in this study.

## Discussion

Social research often yields nominal or ordinal measures which are considered less precise than the interval and ratio measures reported in physical sciences [41]. The English- and Spanish-MFES demonstrated acceptable fit to the Rasch model allowing for the conversion of ordinal responses to logit values. Logits possess several advantages over traditional raw scores. The conversion of ordinal data to equal-interval data means any difference in logits implies equal difference in ability. This allows for a more accurate “location” along the construct [42]. Researchers and clinicians can visualize the order of difficulty or intensity of items and ascertain where a person is located compared to all items. The equal-interval facilitates the assessment of change in scores. For instance, we can see from Table 2 (English) that there is a relatively large gap (distance on latent trait) between *Use steps at home* and *Use public transportation*. This gap of approximately one logit implies construct under-representation. The clinical implication is that interventions aimed at improving confidence at this location may prove less responsive. At the opposite end, a relatively large gap in the construct can be observed between *Answer door/telephone* and *Get dressed/undressed*. While the range of the construct is more limited for the Spanish group, the interval between tasks is much closer, reflecting little to no construct under-representation.

Evaluating the equivalence of psychological measures can facilitate wider application across populations of different languages. We evaluated the factor structure of English- and Spanish-MFES data and found excellent model fit in both languages. Our results support a unidimensional construct of fear of falling among English- and Spanish-speaking older adults who live in urban US populations. Item communalities of the Spanish-MFES exhibited greater variation than the English-MFES items, but were similar to the factor loadings reported by Hill [23]. The unidimensional factor structures obtained from the EFA revealed stronger overall factor loadings with less variation (i.e., English: 0.85–0.97; Spanish: 0.60–0.93) compared to Hill’s two constructs (i.e., indoor activities: 0.40–1.063; outdoor activities: 0.47–0.93).

Our results appear to reinforce Hill’s concern about the limitations of task difficulty in Tinetti’s FES. These findings provided support to conduct an item- and person-level analysis of the English- and Spanish-MFES. There were two items (i.e., *Walk inside house* and *Answer door/telephone*) between the English- and Spanish-MFES that were not within the acceptable range of the core fit statistics. This calls into question the item difficulty range for the observations in this study. Moreover, based on the item reliability results, it may not be possible to discern whether there are significant differences in the estimated measures between items. On the other hand, given the same participants, a “very good” probability exists that persons estimated with high measurements may actually have higher measurements than persons estimated with low measurements. Although the data did not fit perfectly in the Rasch measurement model, the values and differences of the observed and expected variances point to a unidimensional measure of the English- and Spanish-MFES. Notably, although a larger proportion (i.e. 41.4%) of Spanish-speaking participants had less than a high school education, this difference did not appear to influence the group’s ability level overall.

The ceiling effects revealed in Fig. 1 can be partially explained as follows. First, the participants were on average 5–10 years younger than those in the original MFES validation study [23]. Second, a substantial percentage of participants (i.e., 40%) reported to the emergency department for a reason other than falling in the community. These factors combined reflect a sample that could have been higher functioning, thus perceived having the ability to perform activities described in the MFES.

There are many psychometric tools used to measure fear of falling including the FES, the FES revised, the Falls United Kingdom, the Activities-specific Balance Confidence (ABC), the ABC United Kingdom (ABC-UK), and the Confidence in Maintaining Balance Scale [21, 43–47]. Among the limitations of these tools is the lack of translation and cross-cultural validation. Like many of these tools, Hill’s MFES is used widely in other English-speaking countries but was developed and tested in a single study [6]. The combined result of these limitations is the measurement error found across international prevalence reports of fear of falling [6]. This is notable given the positive association between fear of falling and falls, the aging of society, and the incidence of community-based falls [48–50].

### Limitations

The limitation that primarily influenced on our findings was the use of data collected for a study unrelated to the aim of the current study. We acknowledge that including adults 55 years of age or older could influence the results because of higher physical functioning levels. This age range was used in the parent study because the majority of osteoporotic fractures sustained by females 55 years of age and older can be attributed to a fall [51]. The context of our study procedures was different from Hill’s study. Our participants self-referred to the emergency department of an urban hospital, whereas Hill recruited participants from several community-based settings. Lastly, we acknowledge that the sample size was a limitation to the Rasch measurement method. We caution readers in their interpretation when referring to our findings.

## Conclusion

We explored whether the MFES developed by Hill, then translated in Spanish, was equivalent for English- and Spanish-speakers in the US. We found that the MFES may be a unidimensional measure for both English- and Spanish-speaking older adults. Reducing the MFES from two dimensions to one dimension could advance the practical utility and clinical meaningfulness of a tool for primary care practice to assess an individual’s ability to perform common activities of daily living without falling. Our findings lend support to Hill’s contention that clinical assessment of fear of falling should be based on tasks that induce a great amount of fear in older adults. Large-scale international studies linking the unidimensional MFES to patient outcomes (e.g., decreased activity restrictions and/or falls) will support the validity of this tool for practice. The ultimate goal is to effectively assess fear of falling, minimize activity restriction, and maximize patient outcomes for diverse older adult populations.

## Data Availability

The dataset used during the current study is not publicly available because the data contain potentially identifying or sensitive patient information.

## Abbreviations

MFES: Modified Falls Efficacy Scale
ED: Emergency Department
US: United States
FES: Falls Efficacy Scale
EFA: Exploratory Factor Analysis
WLSMV: Weighted Least Squares Mean and Variance
χ^2^: Chi Square
CFI: Comparative Fit Index
TLI: Tucker–Lewis Index
RMSEA: Root Mean Square Error of Approximation
SRMS: Standardized Root Mean Square Residual
MNSQ: Mean-Square
ABC: Activities-specific Balance Confidence
ABC-UK: ABC United Kingdom
